# Motor Skill Learning of a Racing Video Game With Intuitive versus Inverted and Random Controls

**DOI:** 10.1002/brb3.71682

**Published:** 2026-08-20

**Authors:** Quinn McCallion, Ben Wilhelm, Davin Greenwell, Jacob Feigh, Morgan S. Hoard, Julynne R. Spidell, Brach Poston, Zachary A. Riley

**Affiliations:** ^1^ School of Health and Human Sciences Indiana University Indianapolis Indianapolis Indiana USA; ^2^ Neurologische Klinik und Poliklinik Universitätsklinikum Würzburg Germany; ^3^ Department of Kinesiology and Nutrition Sciences University of Nevada‐Las Vegas Las Vegas Nevada USA

## Abstract

**Aim:**

Motor skill learning relies on increasing proficiency to translate sensory feedback to produce an updated motor command. This experience‐dependent process allows our automatic movements to lead to an expected result—this is known as an “internal model.” Video games provide a structured environment for studying these processes, as they typically develop stable sensory‐motor relationships (or internal models) within the brain.

**Purpose:**

This study examined how disrupting these internal models with two different non‐intuitive joystick control schemes affected short‐term motor learning during a video game task.

**Method:**

Eighty healthy right‐handed adults (males: *n* = 49 and females: *n* = 31) completed three blocks of five timed laps using normal controls (N1), inverted or random controls (IC/RC), and then returned to normal controls (N2). Half the subjects used inverted controls, while the other half used random controls during the IC/RC block. Performance was measured through lap times, and error was manually quantified by recording wall contacts.

**Finding:**

Results showed that all participants improved from lap 1 to 5 within each testing block. The inverted control group had significantly slower initial lap times and more wall contacts during the first lap of the IC/RC and N2 blocks. There was a trend (*p* = 0.06) for the IC group to improve more than the RC group from lap 1 to lap 5 in the IC/RC block. Additionally, for the IC group, the first lap of block 3 was significantly slower than where they left off before the IC block (*p* = 0.002). The random control condition did not experience this same increase in lap time and wall contacts during lap 1 of the N2 testing block. Instead of experiencing anterograde interference, the random control group may have been subject to de novo learning taking place.

**Conclusion:**

This study therefore highlights the flexibility of motor learning systems and supports the accompaniment of interference with motor adaptation.

## Graphical abstract

This study examined how disrupting these internal models with two different non‐intuitive joystick control schemes affected short‐term motor learning during a video game task. Instead of experiencing anterograde interference as the inverted control group did, the random control group may have been subject to de novo learning taking place.

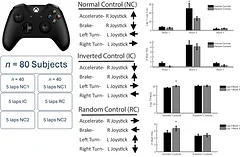

## 1 Introduction

Motor skill learning is a dynamic process that relies on repeated task execution, paired with sensory feedback to improve task performance and automaticity (Krakauer et al. 2019). Through repeated practice of a movement, the brain forms and refines internal models, or an experience‐dependent automatic understanding of how our movements (like pressing a button or tilting a joystick) lead to expected results (Kawato and Wolpert, 1998; Lonini et al., 2009). These neural representations are shaped and refined by consistent associations between motor commands and the outcomes they produce (Shadmehr and Krakauer 2008; Wolpert et al. 1995). For example, in the context of standard video game play, at a young age players internalize the relationship between tilting the joystick forwards to accelerate and tilting back on the joystick to reverse or brake. With continuous exposure to this standardized control scheme throughout life, sensorimotor associations are reinforced and become automatic, allowing for skilled performance with reduced attentional demands (Makino et al. 2016; Yang et al. 2022).

Error feedback learning is thought to rely on sensory prediction errors, where the expected result and actual result do not match (Albert and Shadmehr 2016). During the early stages of motor learning, when internal models are not fully developed, error‐based learning can be important in further integrating and stabilizing the task within the motor cortex (M1), posterior parietal cortex, and cerebellum (Desmurget et al. 2001; Ikegami et al. 2022). Additionally, the degree of learning (rate and magnitude) can depend largely on the magnitude of the error feedback (Ahmadi‐Pajouh et al. 2012; Albert and Shadmehr 2016). In the context of video game play, particularly for racing games, veering off the track or hitting a side wall may constitute an error. The magnitude of the error can vary and depend on the speed of the car and the angle of impact with the wall, resulting in a scalable amount of time lost. Integrating these signals in the specific brain regions will result in reduced future errors and improve performance efficiency (Krakauer and Mazzoni 2011).

Video games are a valuable and practical tool for studying motor learning. They provide structured, goal‐directed tasks with built‐in feedback and motivational reward systems, facilitating engagement and consistent task repetition (Green and Bavelier 2003; Palidis et al. 2019). An additional benefit of video game design is that they are usually built around an intuitive control scheme using approximated physics engines to allow users to quickly adapt to the gameplay (Mitko et al. 2024; Ullman et al. 2017). While video games often offer the ability to change the gameplay controls, the only prevalent alternative strategy that is used is inverting the Y‐axis, which may arise from games with flight controls or personal preference (Frischmann et al. 2015). Because video game control schemes tend to be standardized and intuitive, they promote the development of highly stable internal models (Kawato and Wolpert 1998). Non‐intuitive control schemes—such as those with altering joystick directions—introduce a conflict between previously learned associations and the new, novel demands of the task (Yang et al. 2021). This can lead to interference (retrograde or anterograde, depending on task) that can slow the learning process (Krakauer et al. 2005; Krakauer et al. 2006; Sing and Smith 2010). In such cases, successful performance requires suppression of habitual motor commands and engagement in the updating of sensorimotor mappings, likely involving de novo learning (constructing and not adapting a new controller from scratch), subsequent error‐driven adaptation, and possible recruitment of alternate neural circuits (Haith et al. 2022; Taylor et al. 2014). Then, transitioning back to the original task results in a switching cost, making the performance of the original task slower and more error‐prone (Monsell 2003; Vandierendonck et al. 2010).

This study investigates how participants adapt to task switching between intuitive and non‐intuitive controls in a racing video game. Specifically, traditional internal models of normal joystick control (e.g., joystick movement left, kart goes left) were replaced with inverted controls (e.g., joystick movement right, kart goes left) and random controls (e.g., joystick movement right, kart accelerates) in an A_1_‐B‐A_2_ testing paradigm. This manipulation could theoretically disrupt participants’ pre‐existing internal models and force them to override previous associations to build new ones. By examining performance metrics, our goal was to explore the flexibility of motor learning systems, particularly how internal models may be constructed or disrupted when habitual motor strategies become maladaptive.

## 2 Methods

A total of 80 healthy, right‐handed adults (males: *n* = 49 and females: *n* = 31) between the ages of 18–45 years old, with no history of significant neurological or physical disorders that would interfere with their ability to complete the task, were recruited to take part in this study. Participants were asked to abstain from caffeine or stimulants (i.e., coffee, pre‐workout supplements, soda, energy drinks, tea, nicotine, or tobacco products, etc.) for at least 12 h before their visits.

All participants were given a copy of the informed consent form, which was explained to them by the researchers. Participants were asked to thoroughly read through the informed consent form and to ask any questions that they might have before signing. All procedures were reviewed and approved by Indiana University's Internal Review Board (Protocol #20694) and conducted in accordance with the Declaration of Helsinki.

The study utilized a comparative between‐group design to study learning effects while playing an open‐source racing video game (SuperTuxKart). We did not record the amount of previous gaming experience among the subjects, though it was confirmed that none had played SuperTuxKart previously. Subjects played the game using a customized dual joystick control scheme with a standard Xbox One wireless controller (Microsoft). SuperTuxKart is an open source “kart racing” video game where players must navigate a kart through a complex, 3D racetrack as quickly and accurately as possible to achieve faster lap times. Performance in SuperTuxKart is quantified as the time to complete one full race lap. Accurate steering and appropriate use of acceleration/braking results in faster lap completion, shorter race times, and better performance.

The game's code was modified to make the karts faster, heavier, and harder to steer within the internal physics engine of the game. The track used for this study was an enclosed tunnel with no alternate routes or shortcuts available. To help ensure that learning was organic, researchers refrained from commenting on subjects’ performance or providing detailed instructions. To keep the subjects engaged, researchers reminded them to “continue focusing on strategies for improvement” after every other lap. Finally, to maintain consistency, the same racetrack and kart were used across all practice blocks for all subjects.

**FIGURE 1:**
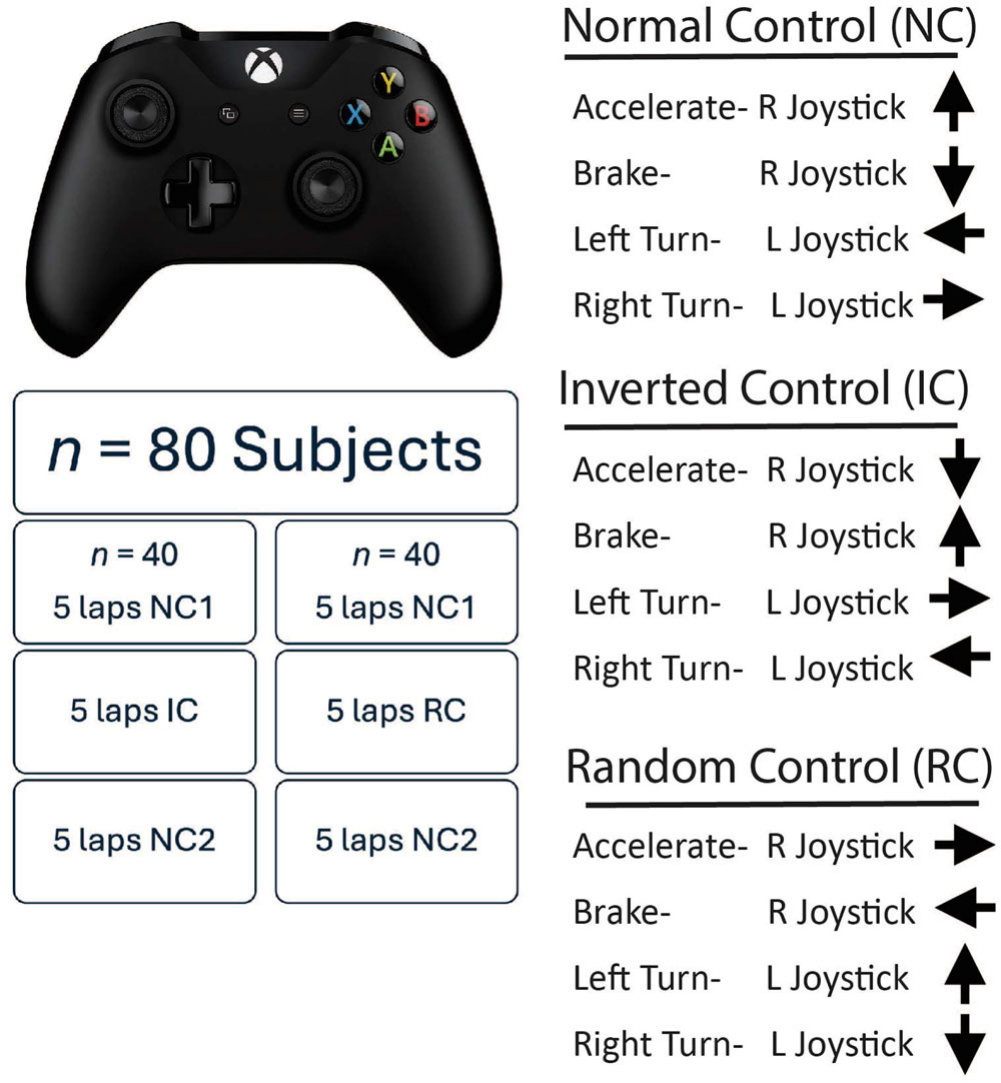
Shows the controller used for the gameplay. On the bottom left is the description of the subjects and experimental design. On the right is a depiction of the control schemes used with the controllers. For reference, the normal controls are what would be expected for most videogames.

Single lap time trials were performed on a closed track with no computer or human‐controlled opponents. Subjects were only able to access steering, acceleration, brake, and reverse functions, and the game's “power‐up” features (like turbo boosts) were disabled. Subjects were counterbalanced and randomly assigned to two groups: (1) *normal* controls and *inverted* controls (n = 40), or (2) *normal* controls and *random* controls (n = 40). After a single familiarization lap on the track with the normal controls, subjects performed 3 blocks of 5 laps with 30 s of rest between each lap in an A_1_‐B‐A_2_ testing paradigm. The first block (A_1_) was using the normal controls (NC1) for both groups. The second block (B) required the subjects to race with inverted controls (IC) or random controls (RC), depending on the group they were randomized to. Finally, the third block (A_2_) switched back to the normal controls again (NC2). A timeline of the experiment is depicted in Figure 1.

For the *normal* control scheme, the left joystick was used to steer left and right by pressing left or right, respectively, while the right joystick was used to accelerate by pressing up (forward) on the joystick; braking and reverse were initiated by pulling/holding down (backward) on the joystick. The *inverted* control scheme consisted of an axis inversion, where the left joystick was used to steer left and right by pressing right or left, respectively, while the right joystick was used to accelerate by pressing down on the joystick, braking and reverse were initiated by pressing up on the joystick. The *random* control scheme consisted of a fixed‐axis reassignment with randomly generated controls that were held constant throughout the middle training block; the left joystick was used to steer left and right by pressing up and down, respectively. The right joystick was used to accelerate and brake by pressing the joystick right and left, respectively. The controls for the intuitive and non‐intuitive conditions are depicted in Figure 1.

The two main outcome variables from the study were the individual lap times and the number of times the kart contacted the wall. Since ‘wall contacts’ were not a direct output from the game itself, these were manually observed and logged in a separate file by an investigator. All analyses were performed separately on the lap time and wall contact data. Initially, the data was compared with a 2 groups (inverted/random control) x 3 blocks (NC1, IC/RC, and NC2) x 5 laps mixed model ANOVA for each lap time in seconds and the number of wall hits. As we expected, there was a significant effect of lap time across the five laps in each block, as will be presented in the results. Due to this, we also calculated the difference between lap 1 and lap 5 within each block in absolute time (seconds) and wall hits and tested that data with a 2 groups (inverted/random control) x 3 blocks (NC1, IC/RC, NC2) mixed model ANOVA. To examine the effect that the intermediate block had on normal control gameplay, we compared the lap times and number of wall hits for the fifth lap of NC1 to the first lap of NC2 with a paired t‐test.

All statistical analyses were performed in SPSS, and significance was noted with *p <* 0.05. Tukey's post‐hoc tests was used, where appropriate. Normality of the outcome distributions were assumed based on sample size and the robustness of a mixed model ANOVA to moderate non‐normality with identical group subject numbers.

## 3 Results

**FIGURE 2:**
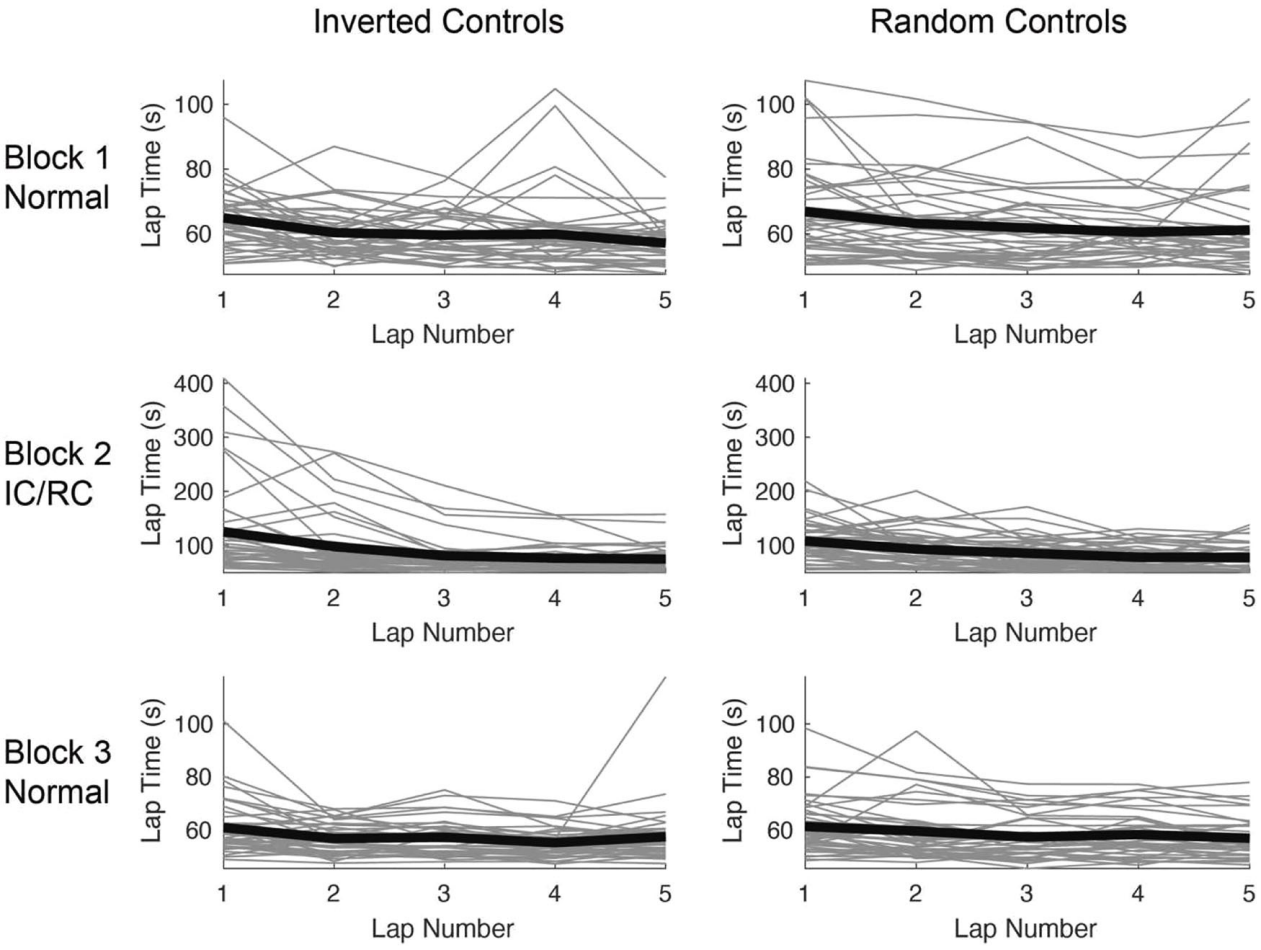
Represents the lap times across each of the 5 laps in the 3 blocks for the inverted and random control groups. The thin grey lines represent the individual responses for each subject, while the thick black line represents the mean of the entire group. Lap times are in seconds.

**FIGURE 3:**
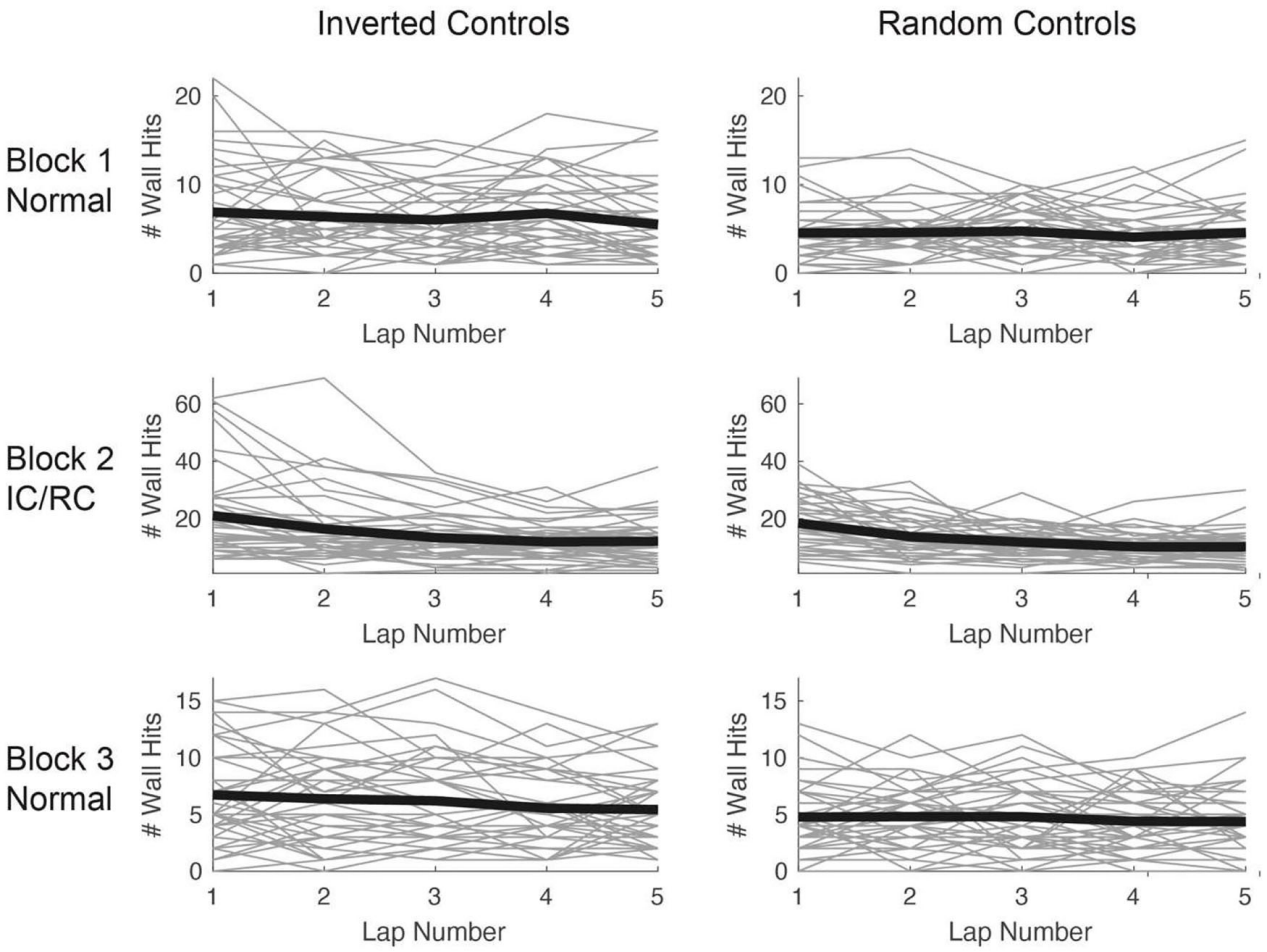
Represents the number of wall hits across each of the 5 laps in the 3 blocks for the inverted and random control groups. The thin grey lines represent the individual responses for each subject, while the thick black line represents the mean of the entire group. Wall hits were manually recorded by the experimenters each time the subject contacted the wall of the enclosed race track.

With the 2 group x 3 block x 5 laps mixed‐model ANOVA examining lap times, there were main effects of the testing block, with the IC/RC block having longer lap times (*F* [2, 156] = 79.943*, p* < 0.001). There was also a main effect of lap number, as all groups got progressively faster from lap 1 to lap 5 in each block (*F* [4, 312] = 46.927*, p* < 0.001). As expected, there was an interaction between block and lap number (*p <* 0.001) and even block, lap number, and group (*p =* 0.024), but there were no stand‐alone between‐subjects effects of the IC/RC groups (*F* [1, 78] = 0.009*, p* = 0.925). With the same 2 group x 3 block x 5 lap mixed‐model ANOVA examining the number of wall hits, there was a similar main effect for block (*F* [2, 156] = 153.841*, p* < 0.001) and lap number (*F* [4, 312] = 38.455*, p* < 0.001), as well as an interaction between the two (*p <* 0.001). Different from the lap times, there was a trend for a significant difference between the IC/RC groups in the number of wall hits, with the IC group hitting the wall more frequently (*F* [1, 78] = 3.783*, p* = 0.057). However, this did not result in a significant interaction across block, lap number, and group (*p =* 0.952). All the individual results for the lap times and number of wall hits across groups and blocks are shown in Figures 2 and 3.

**FIGURE 4:**
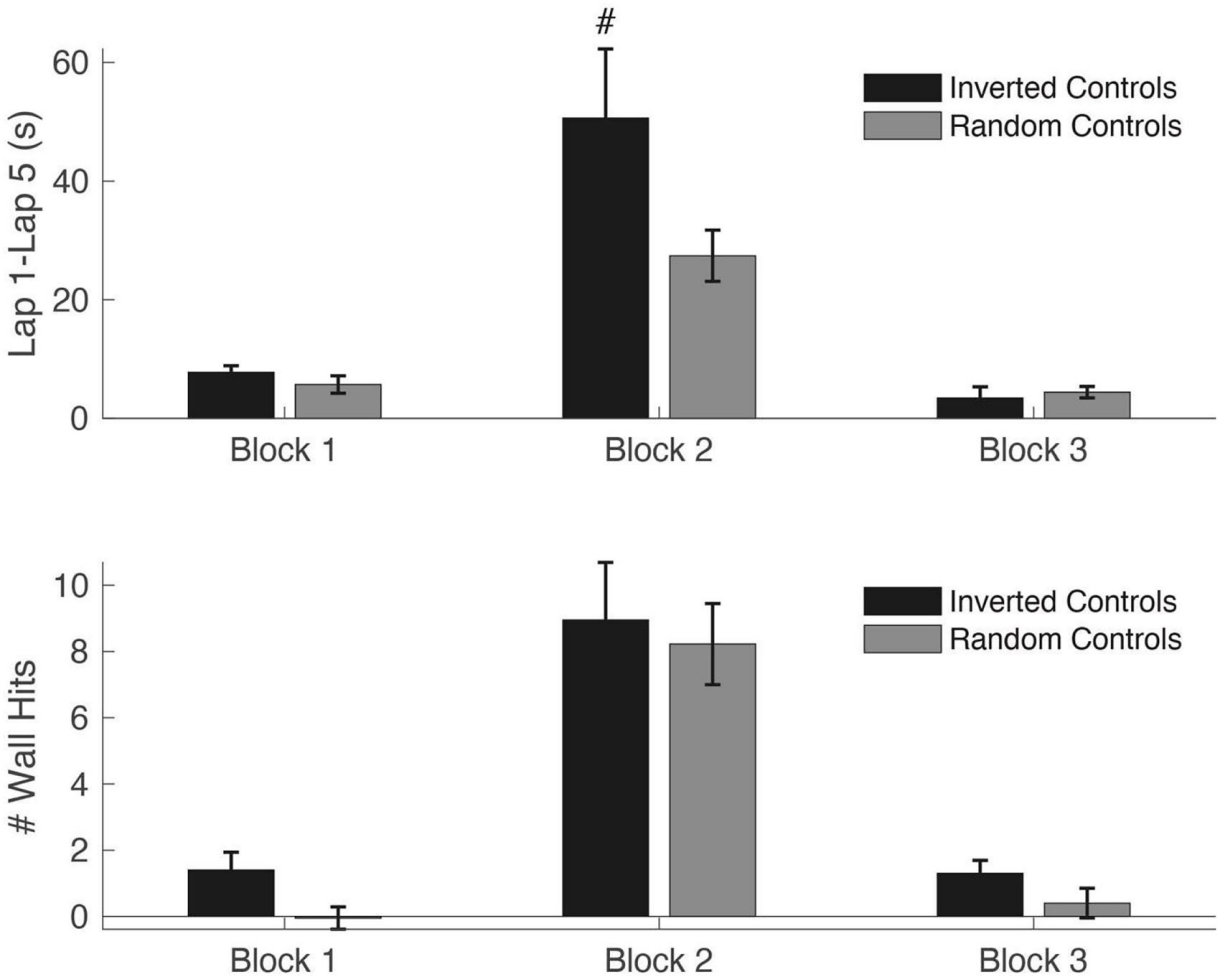
Top‐ The difference in time (s) between laps 1 and 5 across the three blocks for the inverted (black bars) and random (grey bars) controls. The change in lap time within block 2 approached significance between the two groups (#*p* = 0.06) Bottom‐ The difference in the number of wall hits between laps 1 and 5 across the three blocks for the inverted (black bars) and random (grey bars) controls.

Given the extreme variability across the lap numbers and blocks for the two groups, we did additional analyses to explore specific relationships. Since there was a significant effect of lap time across the five laps in each block, we examined the difference between lap 1 and lap 5 within each block in absolute time (seconds) and wall hits. There was a main effect for block when comparing lap 1 to lap 5 times (*F* [2, 156] = 29.029*, p* < 0.001), but more importantly, there was a block x group interaction (*p* = 0.039) and a trend for a significant between‐subject group effect (*F* [1, 78] = 3.458*, p* = 0.060), suggesting that the change in lap time within a block was greater for the IC group (Figure 4, top) and that by reducing the variability of the data (intermediate laps within a block), the data became closer to showing a between‐subject group effect. There was a similar main effect for block when comparing the number of wall hits from lap 1 to lap 5 (*F* [2, 156] = 47.909*, p* < 0.001), but there was no block x group interaction (*p* = 0.920) or between‐subject effect of group (*p* = 0.200, Figure 4, bottom).

**FIGURE 5:**
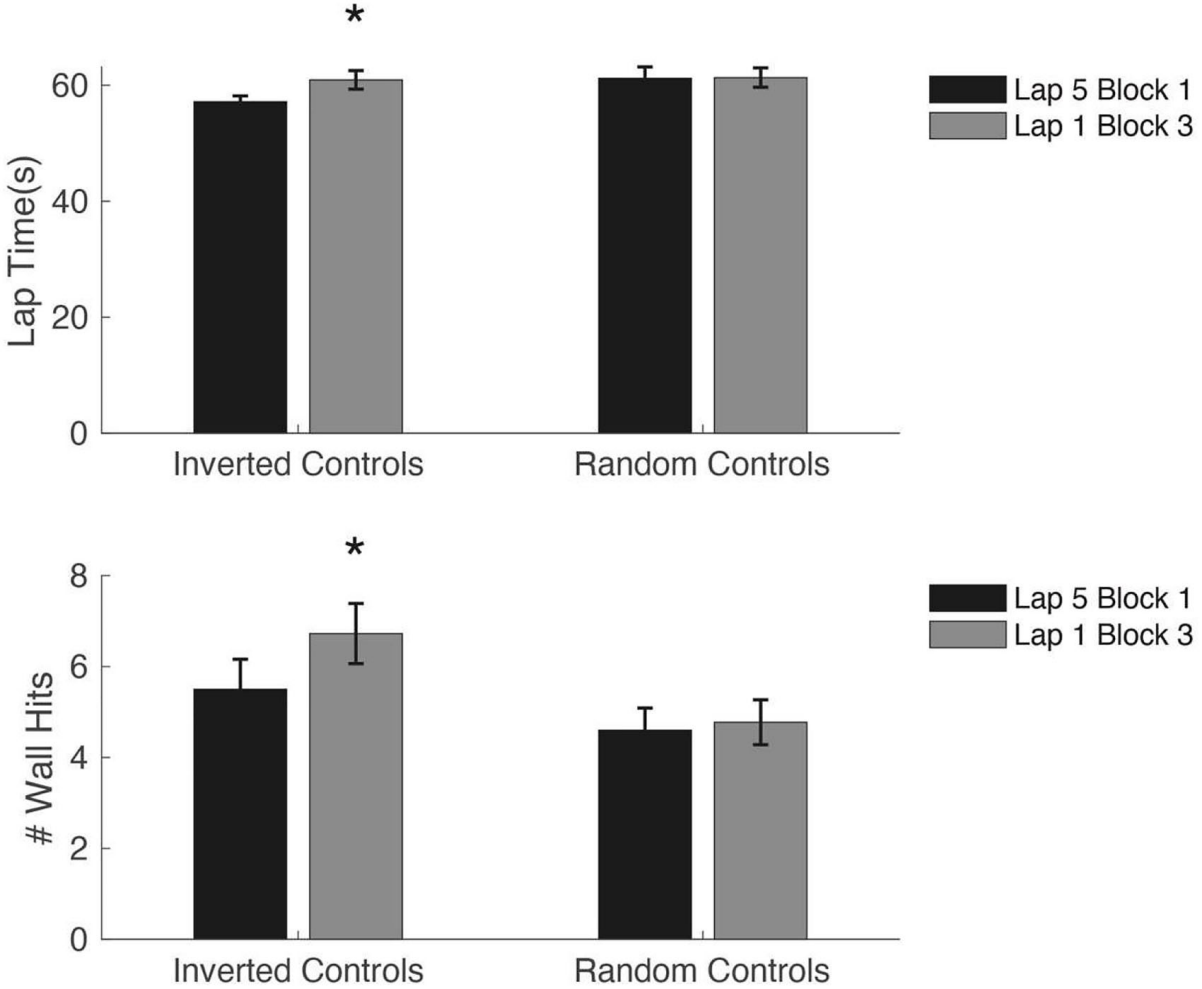
The last lap of NC1 compared to the first lap of NC2, with the inverted or random control groups. The top panel shows the lap times and the bottom panel displays the number of wall hits. In both cases, the inverted control group was significantly worse (*p < 0.05) when returning to normal controls during the first lap of NC2.

Additionally, we compared the last lap of block 1 to the first lap of block 3 (2 groups x 2 time points) to examine the lingering after‐effects of the IC and RC blocks between the NC (NC1 & NC2) practice blocks. For the lap times, there was a main effect for time (*F* [1, 76] = 5.250*, p* = 0.025; Figure 5, top), and more importantly, there was a significant interaction between the groups and lap times (*F* [1, 76] = 4.605*, p* = 0.035), but no between‐subject difference (*F* [1, 76] = 1.010*, p* = 0.318). This can be interpreted that the two groups were not different overall, but the changes in lap time between the last lap of block 1 and the first lap of block 3 were different for the two groups. Specifically, for the IC group, pairwise comparisons showed that the first lap of block 3 was significantly slower than where they left off before the IC block (*p* = 0.002). Alternatively, there was no difference between the last lap of block 1 and the first lap of block 3 for the RC group (*p* = 0.918). We then applied the same analysis to the number of wall hits, and there was again a main effect for time (F[1, 76] = 4.263*, p* = 0.042; Figure 5, bottom), though the interaction was not significant (*F* [1, 76] = 3.621*, p* = 0.061). There was nearly a between‐subject difference in the number of wall hits (F[1, 76] = 3.889*, p* = 0.052).

## 4 Discussion

This study aimed to investigate how short‐term motor learning is affected when an established internal model is disrupted by replacing intuitive video game controls with two different non‐intuitive control schemes: one with inverted controls and one with completely random controls. As expected, within each block, subjects got faster and more accurate (less wall hits) across the 5 laps of practice. Also as predicted, the IC/RC block had much slower lap times and more wall contacts as subjects switched control schemes. However, there were no differences observed between the IC and RC groups in either of the outcome variables. When examining the difference in lap times and wall hits between laps 1 and 5 within each block, there were differences, with the IC group showing the biggest change during the IC/RC block. The aftereffects of the IC/RC block appear to be higher in the IC group, as they were slower and made more wall contacts with the standard controls in lap 1 of NC2 than they did in lap 5 of NC1. Taken together, these results indicate that the inverted controls, which are most closely related to the original controls, are more difficult to learn initially, and are potentially disrupting the motor program the most when switching back to normal. The remainder of the discussion will present some explanations for why this finding occurred.

The IC group was slower and had more wall contacts than the RC group in lap one of the IC/RC blocks. This may be due to participants in the IC group experiencing anterograde interference at the beginning of the IC block, introduced through practice of the normal controls in N1 (Sing and Smith 2010). Anterograde interference commonly occurs with an A1‐B1‐A2 experimental model (Brashers‐Krug et al. 1996). Studies including two “normal” practice blocks (A1 and A2) separated by the performance of the inverse task (B1) can result in the diminished performance of the inverse task at the beginning of the B1 block. This is in line with current work by Brawn et al. (2018), Shadmehr et al. (1994), Yang et al. (2025), and Lee et al. (2022), who observed the same anterograde interference with inverse task paradigms. Within the current study, it is likely that during the transition from the normal controls in N1 to the inverse control in the IC block, the same internal model would be used and updated. However, due to the practice of normal controls increasing the stability of the internal model during N1, there was a greater task switching cost that resulted in an increase in the amount of anterograde interference present at the beginning of the IC block (Mooney et al. 2021). Anterograde interference can be characterized by two factors: a slower rate of learning of the inverse task and an increase in initial task error (Krakauer 2009; Lee and Schweighofer 2009; Sing and Smith 2010). These two characteristics can be observed independent of one another and therefore can make it difficult to quantify the total volume of interference that may have occurred (Sing et al. 2009). For example, we observed both an initial increase in wall contacts and lap times (error) in the first lap of the IC block for the IC group, and although a weaker measure, we observed an absolute increase in performance from laps 1–5 of the IC/RC block in the IC group. These results should be interpreted cautiously but suggest anterograde interference was present between N1 and IC blocks. However, even with anterograde interference present at the beginning of the IC block, improvements in performance between laps 1–5 of N1, IC, and N2 were observed within the IC group. This indicates that with relatively little practice, there is the potential for relatively large performance improvements, despite the presence of interference (Karni et al. 1998).

Subjects in the IC group were significantly slower and had more wall contacts in lap one of N2 when compared to their own lap five in block N1. While it is common to experience a decrease in performance with anterograde interference between the A1 and B1 sections of an A1‐B1‐A2 model, participants can also experience anterograde interference between B1 and A2 (Fu and Santello 2015). This phenomenon is particularly common with the use of an inverse control model where practice of the inverse condition can disrupt the motor memory of the normal controls (Escalante and Lei 2023). Nepotiuk and Brown (2022) determined that when an inverse chopping condition was performed in close proximity to the normal task, it could destabilize the normal task's motor trace. However, the authors also note that the extent of this destabilization depends on the expertise of the participant, how well learned the internal model for the normal controls is, and the ability of the participant to deploy an internal model based off of the contextual information of the task. During the performance of the IC block, the short time interval between the performance of the normal controls and similarities in the task dynamics between the normal and inverse controls, although not physiologically measured, may have led to updates of the same internal model (Shadmehr and Mussa‐Ivaldi 1994). As a result, anterograde interference may also be imposed when beginning the N2 block. During the IC block, adaptation to the inverted controls may have resulted in a greater task switching cost when going back to normal controls during N2 (Kessler et al. 2009). Adaptation to the inverted controls, although speculative, may have also impeded feedforward command at the beginning of N2. Feedforward control allows for a predictive model of the desired motor output to be used for more proficient execution of a motor task (Maeda et al. 2018). Delays in accurate feedforward control can contribute to inefficient motor output (Wagner and Smith 2008). Together, greater task‐switching costs as a result of anterograde interference and altered feedforward control due to adaptation of a shared internal model, may both contribute to the initial decrease in performance at the beginning of N2 (Bustos et al. 2024).

Conversely to the IC group, there was no significant difference between the final lap of N1 and the first lap of N2 for the RC group. This suggests the same anterograde interference effects may not be present. While factors such as lower power or higher variability during this block may have contributed to the observed outcomes, it may also be plausible that the performance of the random controls elicited de novo learning and employed the use of a different internal model to complete the task (Yang et al. 2021). This supports some of the prior research on dual‐adaptation paradigms, where separate mappings are learned for different contexts within the same environment (Gabriel et al. 2024; Haith et al. 2022; Koch et al. 2018; Yang et al. 2021). The initial decrease in lap time and increase in wall contacts during the RC block may be due to the lingering slow learning process of learning that biased the normal control scheme. However, the fast learning of the new random controls allows for improvements throughout the RC block. Without competition to update the same internal model, “savings” from the normal controls are able to ensure the proficient release of the motor command at the beginning of N2 (Sing and Smith 2010). Due to the potential de novo learning that took place with the random controls and the short retention of the fast learning of the random controls, it is plausible that no delay in switching internal models or task switching costs when returning to the normal controls during N2 would be observed. This highlights how the dynamics of the motor task, even when similar, can have different learning and interfering effects (Chang et al. 2024).

Subjects in the RC group experienced the same trend in decreasing lap times and wall contacts from laps 1–5 within blocks N1, RC, and N2 as the IC group. The variability during the IC/RC block may be due to the need for additional practice to elicit sufficient error‐based feedback and stimulate both the fast and slow processes essential to the learning of a new skill (Sing and Smith 2010). It is plausible that the lap times for the IC/RC blocks (∼70s) when compared to N1 or N2 (∼ >60s) clearly do not represent the actual limit of performance one could expect using these control schemes if sufficient practice were provided; however, it is impossible to determine how much practice is required with the inverse and random controls to reflect the same level of performance as seen within the groups while using the normal controls during N2.

Error‐based feedback has a significant impact on motor learning (Seidler et al. 2013). Despite the lack of direct physiological measures, explicit instruction, or performance feedback from researchers, participants demonstrated improvement in lap times within each practice block. It is likely that this improvement is driven by internal error detection mechanisms triggered by the observation of their wall contacts and lap times (Wen and Haggard 2020). This aligns with evidence suggesting that the motor cortex adjusts output based on perceived performance errors, even in the absence of external correction (Gabitov et al. 2020), and supports a simple two‐process learning model proposed by Sing and Smith (2010) consisting of slow and fast components. The fast component adapts quickly and responds rapidly to motor error; however, has a small capacity for retention. As skill performance improves and errors reduce, the error‐driven learning will also reduce and will dampen the contribution of the fast process to learning. The slow process seeks to mitigate these losses in learning by adapting and responding more gradually to motor error so that it can become the major contributor to learning when the fast process plateaus. The slow process of learning is impacted by task duration and stabilization, factors that can also determine the degree of anterograde interference, therefore suggesting these two processes may be linked. Within the different practice blocks, learning took place. However, through the use of a task‐switching paradigm, the execution of a directly inverted control scheme may have created uncertainty within the internal model for the normal controls, leading to greater instability error in the motor program at the beginning of the IC/RC and N2 blocks, therefore increasing potential for error‐based learning within the IC group (Crevecoeur et al. 2010). On the other hand, completely random controls may vary enough in task dynamics to utilize a different internal model, therefore having less impact on the strength of the motor memory for the normal controls and reducing the total volume of error‐based learning taking place (Yang et al. 2021).

Practically, these findings highlight the usefulness of video games as controlled, repeatable environments for probing motor learning and internal model formation. The ability to precisely manipulate control schemes, task difficulty, and feedback could provide a unique advantage in the study of neuroplasticity and interference learning (Mishra and Gazzaley 2014). Better understanding of interference learning also has potential implications for rehabilitation, where relearning, modifying, or establishing new internal models is often necessary due to injury or disease. Games with adaptive or progressively altered control schemes could provide novel platforms for engaging individualized therapy. Careful consideration should, however, be given to the specific task dynamics so that interference can be minimized. Variables such as education level, cognitive performance, and previous experience were not collected, and therefore, their effects on learning should be assessed in future studies. In the present study, these factors may have contributed to undetected baseline differences between groups and, consequently, to the between‐group differences reported. Also, wall contacts were manually logged by the same researcher and could have also been a limitation. However, with no differences detected between groups after any of the testing blocks, it is likely these factors did not affect the outcomes of the study.

## 5 Conclusion

In conclusion, this study demonstrates that short‐term exposure to inverse and random video game controls can have different effects on the internal model for the normal controls. Inverse controls may pose a greater perturbation to the internal model of the normal controls, and this is likely due to the closely integrated task dynamics, giving rise to anterograde interference. These findings contribute to our understanding of motor adaptation and highlight video games as a valuable tool for studying sensorimotor learning in applied settings.

## Author Contributions

**Zachary A. Riley**: conceptualization, investigation, Writing – original draft, Writing – review and editing, methodology, validation, formal analysis, project administration, supervision. **Jacob Feigh**: conceptualization, methodology, investigation. **Quinn Mccallion**: conceptualization, investigation, writing – original draft, writing – review and editing, visualization, methodology, formal analysis, project administration, supervision. **Ben Wilhelm**: conceptualization, investigation, validation, formal analysis, writing – original draft. **Morgan S. Hoard**: conceptualization, methodology, investigation, visualization, writing – review and editing. **Julynne R. Spidell**: conceptualization, writing – review and editing, investigation. **Davin Greenwell**: conceptualization, investigation, methodology, validation, visualization, writing – review and editing, writing – original draft. **Brach Poston**: conceptualization, formal analysis, supervision, data curation.

## Funding

The authors have nothing to report.

## Ethics Statement

All procedures were reviewed and approved by the Indiana University's Internal Review board (Protocol #20694) and conducted in accordance with the Declaration of Helsinki.

## Conflicts of Interest

The authors declare no conflicts of interest.

## Data Availability

Data is available upon request.
